# Association between the atherogenic index of plasma trajectory and risk of cardiovascular diseases among hypertensive patients: a prospective cohort study

**DOI:** 10.1186/s40842-026-00302-7

**Published:** 2026-06-12

**Authors:** Weiqiang Wu, Peng Fu, Zefeng Cai, Yuxian Wang, Kuangyi Wu, Hong Zheng, Guanlin Chen, Zekai Chen, Yulong Lan, Yanjuan Chen, Shouling Wu, Youren Chen

**Affiliations:** 1 Department of Cardiology, Second Affiliated Hospital of Shantou University Medical College, 69 Dongxia North RD, Shantou, 515000 China; 2 Department of Endocrinology, Second Affiliated Hospital of Shantou University Medical College, Shantou, China; 3 Department of Cardiology, Kailuan General Hospital, 57 Xinhua East RD, Tangshan, 063000 China

**Keywords:** Atherogenic index of plasma, Hypertension, Cardiovascular diseases, Trajectory analysis

## Abstract

**Background:**

Cardiovascular disease (CVD) remains the leading cause of global mortality, with hypertension and dyslipidemia synergistically exacerbating the atherogenic risk. The atherogenic index of plasma (AIP), calculated as log (triglycerides/HDL-C), reflects lipid-driven atherosclerosis; however, evidence of long-term AIP trajectory patterns and their cardiovascular implications in hypertensive populations remains scarce.

**Methods:**

This longitudinal study included 20,013 hypertensive participants (mean age: 56.8 ± 11.0 years; 81.9% male) from the Kailuan cohort, China. The AIP trajectories (2006–2010) were modeled using latent mixture modeling. Cox proportional hazards models were used to assess associations between AIP trajectories and incident CVD (2010–2020), adjusting for demographics, comorbidities, and medications.

**Results:**

Four distinct AIP trajectories were identified: Low-stable group (*n* = 2,920; AIP range: -0.84 to -0.72), Moderate low-stable group (*n* = 11,121; AIP range: -0.14 to -0.10), Moderate high-stable group (*n* = 5,067; AIP range: 0.42 to 0.53), Elevated-increasing group (*n* = 905; AIP range: 1.12 to 1.46). Over a median of 11.9 (interquartile range 11.3–12.3) years of follow-up, 2,332 CVD events occurred. Compared with the low-stable group, participants in the elevated-increasing trajectory exhibited a 43% higher CVD risk (HR = 1.43, 95% CI:1.14–1.79; *P*-trend = 0.001), with significantly elevated risks of myocardial infarction (HR = 1.80, 1.09–2.51; *P*-trend < 0.001) and ischemic stroke (HR = 1.40, 1.08–1.80; *P*-trend = 0.001). Young hypertensives (< 45 years) with rising AIP trajectories demonstrated a 3.4-fold higher CVD risk (HR = 3.40, 1.03–11.16), whereas no associations were observed for hemorrhagic stroke (*P*-trend = 0.734).

**Conclusions:**

Sustained elevation in AIP trajectories is associated with a higher risk of CVD in individuals with hypertension. Sustained maintenance of optimal AIP levels through regular monitoring, combined with blood pressure control and antihypertensive medication adherence, may reduce the CVD risk in hypertensive populations.

**Graphical Abstract:**

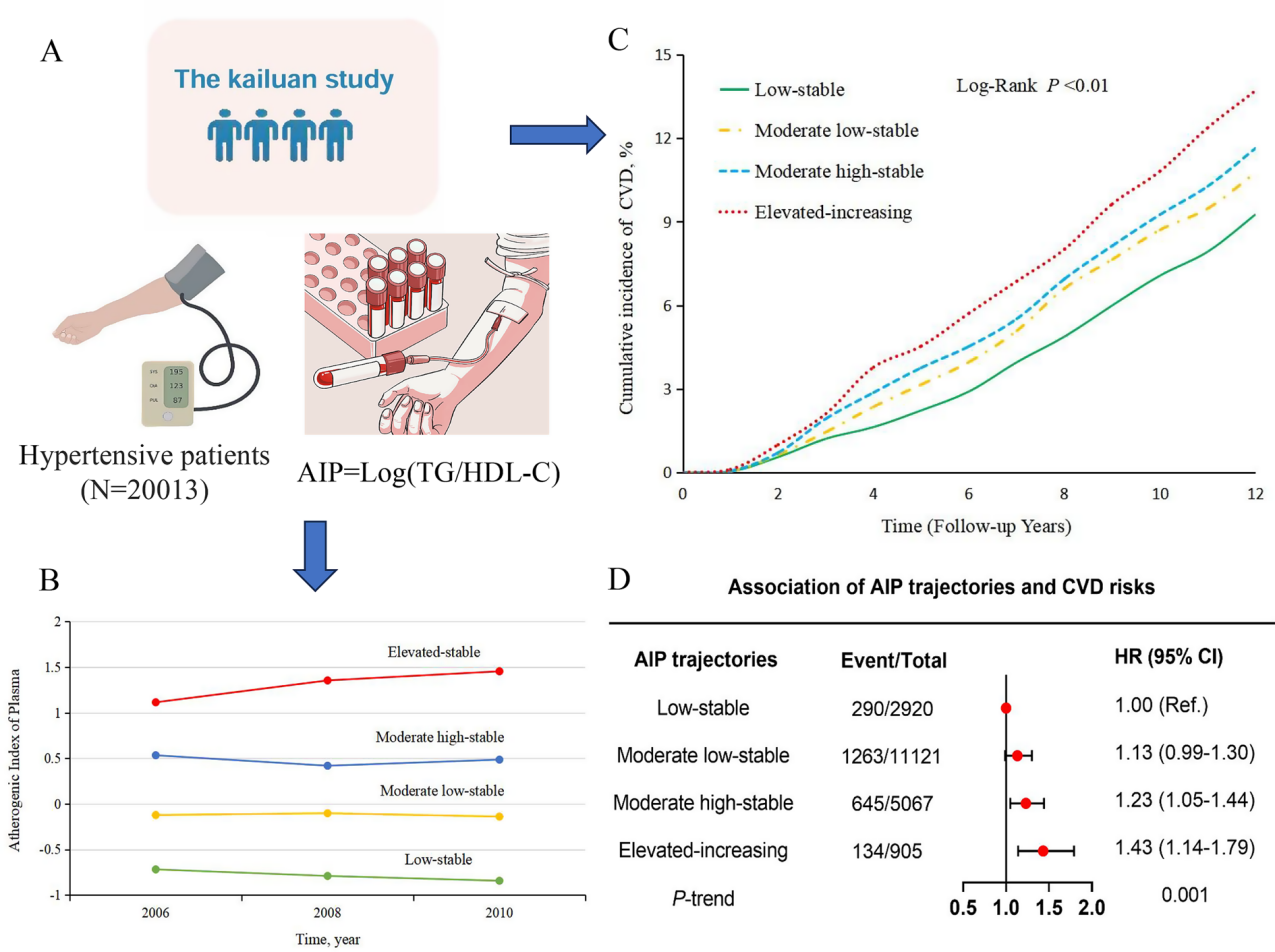

**Supplementary information:**

The online version contains supplementary material available at 10.1186/s40842-026-00302-7.

**Research Insights**

**Table:** 

Section	​Responses
What is currently known?	1. AIP predicts CVD risk in general populations 2. Hypertension amplifies metabolic-cardiovascular interplay 3. Long-term AIP trajectory data lacking in high-risk groups
Key research question?	Do long-term AIP trajectories predict CVD risk in hypertensive patients?
What is new?	1. First 20,013-person cohort tracking 4-year AIP trajectories in individuals with hypertension 2. Sustained elevation in AIP trajectory is associated with a higher risk of CVD in individuals with hypertension.
How might this influence clinical practice?	AIP trajectory analysis refines CVD risk stratification, guiding personalized lipid management in hypertension

## Background

Cardiovascular disease (CVD) remains a leading global public health challenge, characterized by high morbidity and mortality, reduced quality of life, and substantial healthcare costs [1, 2]. Globally, CVD accounts for over 17.9 million deaths annually, with ischemic heart disease and stroke representing the predominant contributors [1, 2]. Hypertension, a major modifiable risk factor, is estimated to affect approximately 1.4 billion adults worldwide, and its prevalence in China has increased dramatically, affecting nearly 30% of the adult population [3, 4]. Hypertensive individuals face a two- to four-fold increased risk of CVD compared to normotensive individuals, and uncontrolled hypertension significantly exacerbates the progression of atherosclerosis and subsequent cardiovascular events [5–7]. Notably, over 60% of hospitalized patients with CVD have a history of hypertension, highlighting the critical need for early risk stratification and targeted prevention strategies in this population [8, 9].

Beyond blood pressure dysregulation, lipid metabolism abnormalities in hypertensive patients further amplify the risk [10]. Dyslipidemia, particularly elevated triglycerides (TG) and reduced high-density lipoprotein cholesterol (HDL-C) levels, accelerates atherosclerotic plaque formation, directly contributing to coronary artery disease, ischemic stroke, and acute cardiovascular events [11]. The atherogenic index of plasma (AIP), calculated as the logarithmic ratio of TG to HDL-C, has emerged as a robust predictor of atherosclerosis and CVD risk [12, 13]. AIP reflects the balance between proatherogenic and antiatherogenic lipid components, with higher values indicating a more atherogenic profile [14]. Epidemiological studies have suggested that elevated AIP levels are associated with an increased risk of myocardial infarction, stroke, and cardiovascular mortality [15]. However, existing evidence predominantly relies on single-time point AIP measurements, neglecting the dynamic nature of lipid profiles over time [16]. Notably, no longitudinal studies have explored the relationship between long-term AIP trajectory patterns and CVD risk in hypertensive populations despite the high prevalence of concurrent hypertension and dyslipidemia.

To address this gap, we utilized data from the Kailuan Study, a large-scale prospective cohort study, to investigate the association between 4 years AIP trajectories and incident CVD among hypertensive individuals. By identifying distinct AIP trajectory groups and their differential CVD risks, this study aimed to provide insights into dynamic lipid monitoring and precision prevention strategies for high-risk hypertensive populations.

## Methods

### Study design and participants

**Fig. 1:**
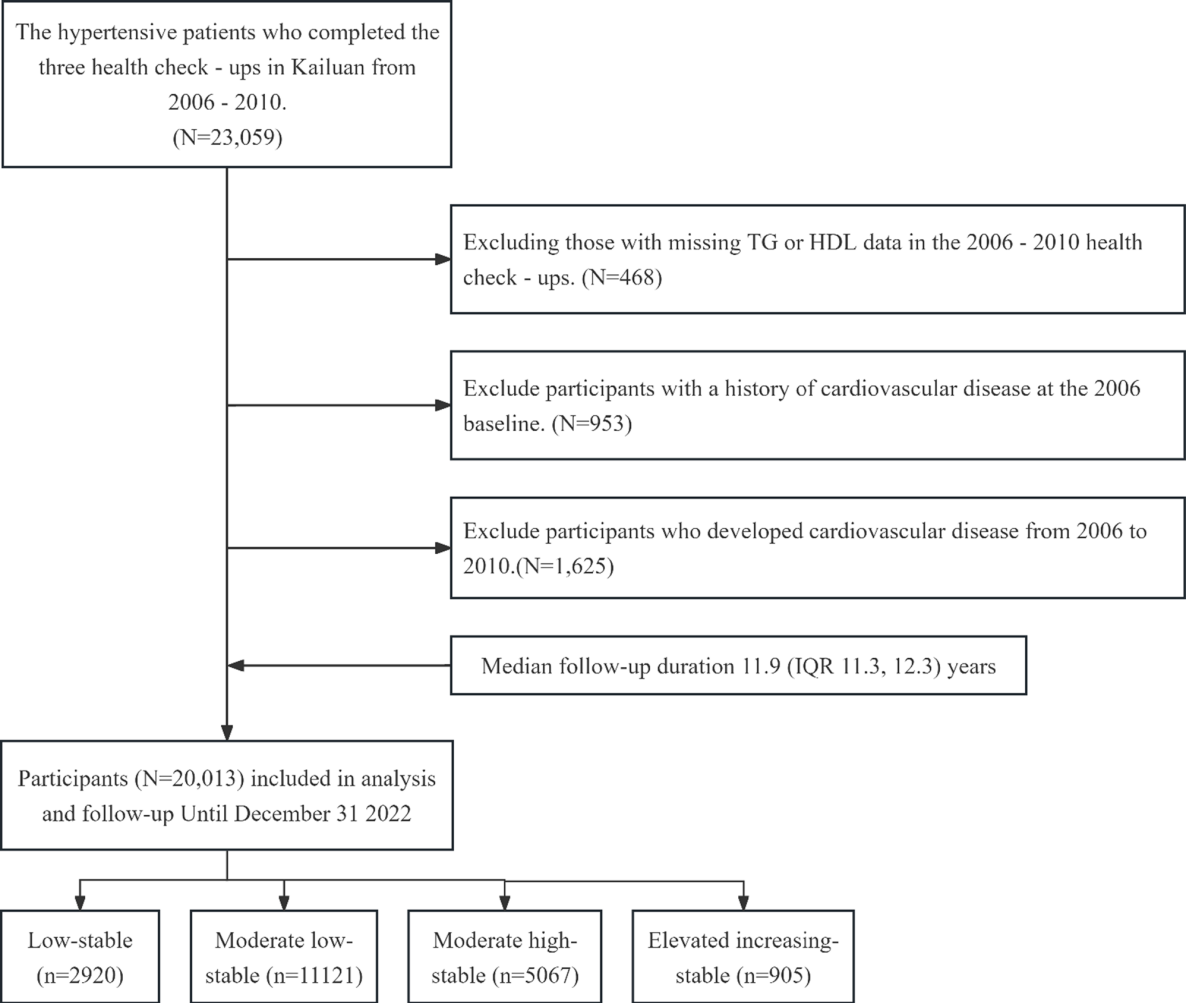
Flow chart for the inclusion of participants in the study

This study utilized data from the Kailuan Study, an ongoing prospective cohort study (registration: ChiCTR-TNRC-11001489) initiated in 2006, involving 101,510 Chinese adults aged ≥ 18 years [17]. Participants with hypertension at baseline (defined as systolic blood pressure [SBP] ≥ 140 mmHg, diastolic blood pressure [DBP] ≥ 90 mmHg, or antihypertensive medication use) were identified [18]. After excluding individuals with pre-existing cardiovascular disease (CVD), cancer, or missing triglyceride (TG)/high-density lipoprotein cholesterol (HDL-C) data, 20,013 hypertensive participants were included in the analysis (Fig. 1). We employed Group-Based Trajectory Modeling (GBTM) and fitted multiple potential models ranging from 2 to 5 groups. By comparing statistical metrics such as the Bayesian Information Criterion (BIC), and comprehensively considering both the clinical interpretability of the groups and the average posterior probability of each trajectory group, the four-group model was ultimately determined to be the optimal model. AIP trajectories were modeled during the exposure period (2006–2010), and participants who developed CVD during this period were excluded. Follow-up for incident CVD outcomes commenced from the end of the landmark period (2010). The study protocol was approved by the Ethics Committee of Kailuan General Hospital (approval number: 200605) and written informed consent was obtained from all participants.

### Data collection and definition

Baseline data were collected using standardized questionnaires, physical examinations, and laboratory tests. All blood samples were collected after an overnight fast of at least 10 h. Lifestyle factors included smoking (current: ≥1 cigarette/day for > 1 year), alcohol consumption (current: ≥100 mL/day for > 1 year), and physical activity (active: ≥3 sessions/week, ≥ 30 min/session). The Body mass index (BMI) was calculated as weight (kg)/height (m²). Blood pressure was measured thrice using a mercury sphygmomanometer and averaged for analysis. Comorbidities Diabetes was defined as fasting plasma glucose (FPG) ≥ 7.0 mmol/L, self-reported diagnosis, or hypoglycemic medication use [19].

### Biochemical measurements and definition of AIP

Fasting blood samples were analyzed for biochemical measurements. Lipid profiles: Total cholesterol (TC), TG, HDL-C, and low-density lipoprotein cholesterol (LDL-C) levels were measured using an automated analyzer (Hitachi 747, Japan). AIP was calculated as the log transformation of the ratio of TG to HDL-C (AIP = log (TG/HDL-C)), with TG and HDL-C expressed in mmol/L. Inflammatory marker: High-sensitivity C-reactive protein (hs-CRP). The AIP index was calculated as the log transformation of the ratio of TG to HDL-C (AIP = log (TG/HDL-C)) as previously described [20].

### CVD outcome ascertainment and follow-up

The primary outcome was incident CVD, defined as the first occurrence of myocardial infarction (ICD-10: I21-I22), ischemic stroke (ICD-10: I63), or hemorrhagic stroke (ICD-10: I61). Outcomes were verified through annual reviews of discharge records from 11 affiliated hospitals supplemented by imaging (e.g., echocardiography, CT/MRI) and biomarker evidence (e.g., troponin) [18]. For participants who experienced two or more events, the timing and type of the first event were recorded as the outcome; however, if a participant experienced different types of cardiovascular events during the observation period, each event was recorded separately. The observation period ended on December 31, 2022, for participants who did not experience any events.

### Statistical analysis

Analyses were performed using the SAS statistical software (version 9.4; Cary, NC, USA). The multiple imputation was employed to handle missing covariate data. Normally distributed continuous variables were expressed as mean ± standard deviation and compared using one-way ANOVA. Skewed continuous variables were presented as median (interquartile range) and analyzed with the Kruskal–Wallis test. Categorical variables were reported as counts (percentages) and compared using the chi-square test. AIP Trajectory Modeling: Latent mixture modeling (SAS Proc Traj) was applied to identify distinct AIP trajectories from 2006 to 2010. The optimal four-group model (low-stable, moderate low-stable, moderate high-stable, and elevated-increasing) was selected based on the Bayesian Information Criterion (BIC). Cumulative CVD incidence across trajectories was estimated using the Kaplan–Meier method, with between-group differences assessed using the log-rank test. The proportional hazards assumption was tested using Schoenfeld residuals, and no significant violations were observed (all *P* > 0.05) [21]. Cox Proportional Hazards Models estimated hazard ratios (HRs) and 95% confidence intervals (CIs) for CVD risk; Model 1: adjusted for age and sex; Model 2: further adjusted for BMI, systolic blood pressure (SBP), LDL-C, hs-CRP, smoking, alcohol use, physical activity, diabetes, and eGFR; Model 3: additionally adjusted for antihypertensive, hypoglycemic, and lipid-lowering medications. Subgroup analyses were stratified by age (< 45 vs. ≥45 years), sex, BMI (< 28 vs. ≥28 kg/m²), diabetes status, and blood pressure control (SBP/DBP < 140/90 vs. ≥140/90 mmHg). Sensitivity analyses: CVD events within 2 years were excluded to mitigate reverse causality; robustness was tested by excluding users of antihypertensive, lipid-lowering, or hypoglycemic agents at baseline; adjusted for both baseline (2006) and endpoint (2010) AIP levels; excluded participants with secondary hypertension; excluded participants who newly initiated statin therapy during the follow-up period; excluded participants with missing TC and LDL-C data at baseline; excluded participants with AIP values in the highest and lowest 0.5% (extreme outliers); included SBP, FBG, BMI, and LDL as time-dependent covariates during the follow-up period. Statistical significance was set at a two-tailed p-value < 0.05.

## Results

### AIP trajectories and baseline characteristics

**Fig. 2:**
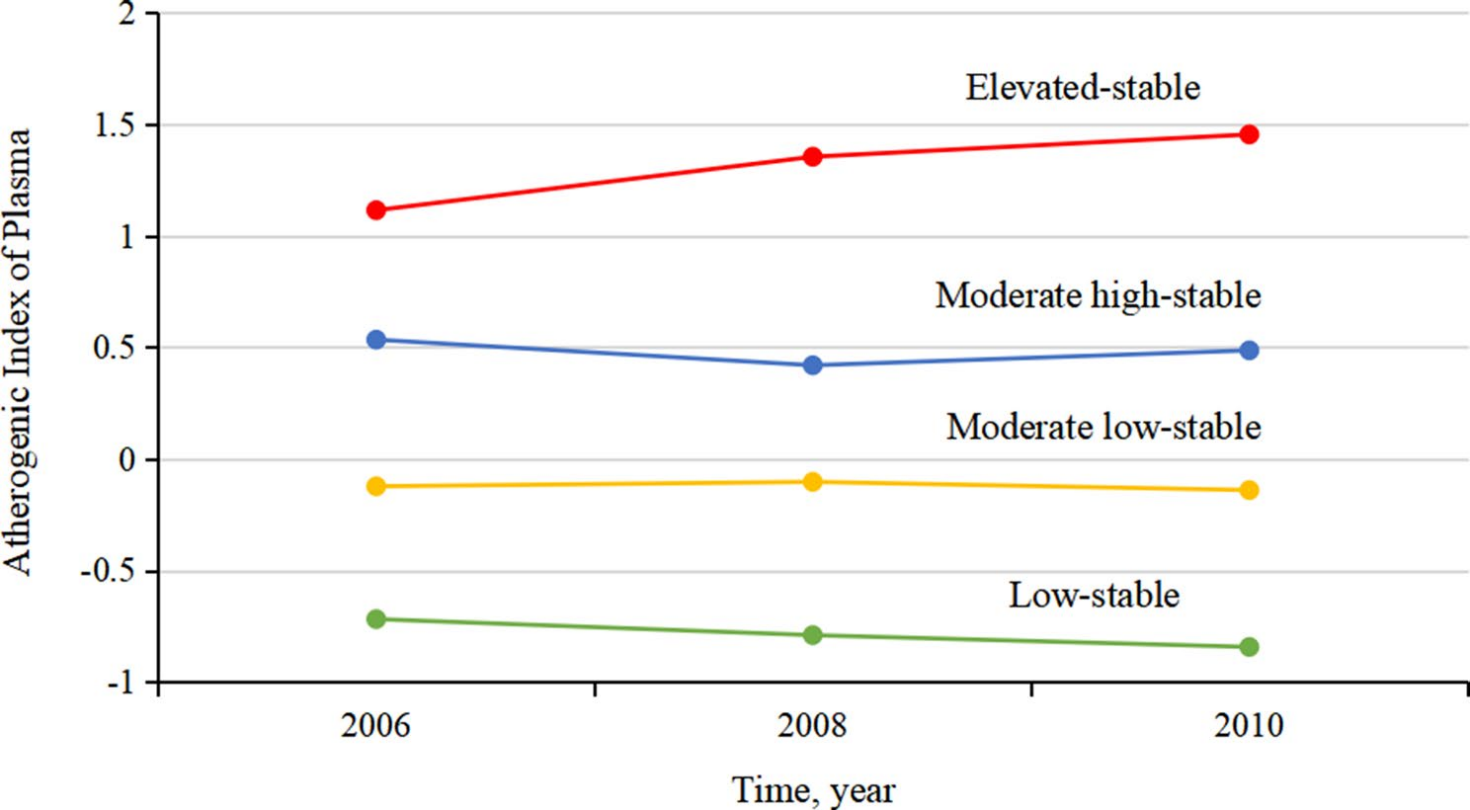
Atherogenic index of plasma trajectories in hypertensive patients during 2006–2010

A total of 20,013 hypertensive participants were included in the analysis, with a mean age of 56.79 ± 10.98 years, and 81.9% were male. Four distinct AIP trajectories were identified over the 4-year follow-up (Fig. 2): Low-stable group (*n* = 2,920; AIP range: -0.84 to -0.72), Moderate low-stable group (*n* = 11,121; AIP range: -0.14 to -0.10), Moderate high-stable group (*n* = 5,067; AIP range: 0.42 to 0.53), Elevated-increasing group (*n* = 905; AIP range: 1.12 to 1.46). Compared to the low-stable group, participants in the elevated-increasing group were younger (53.14 vs. 59.80 years), predominantly male (86.2% vs. 80.7%), and exhibited higher rates of adverse metabolic profiles and lifestyle factors, including elevated BMI (27.25 vs. 24.14 kg/m²), triglycerides (4.88 vs. 0.74 mmol/L), fasting plasma glucose (6.60 vs. 5.56 mmol/L), and lower HDL-C (1.19 vs. 1.91 mmol/L; all *P* < 0.01). Additionally, this group had a higher prevalence of diabetes (29.8% vs. 9.0%), smoking (47.8% vs. 33.3%), and alcohol consumption (46.5% vs. 31.7%), as well as increased use of antihypertensives (39.2% vs. 26.2%) and lipid-lowering drugs (2.98% vs. 0.45%; all *P* < 0.01; Table 1).

**Table 1:** Baseline characteristics of hypertensive patients according to the trajectories of AIP from 2006 to 2010

Variables	Total	Low-stable	Moderate low-stable	Moderate high-stable	Elevated-increasing	*P* value
Participants	20,013	2920	11,121	5067	905	*/*
Age (years)	56.79 ± 10.98	59.80 ± 10.75	57.05 ± 11.07	55.13 ± 10.63	53.14 ± 9.75	< 0.01
Male, N (%)	16,397 (81.9)	2355.0 (80.7)	9025.0 (81.2)	4237.0 (83.6)	780.00 (86.2)	< 0.01
BMI (kg/m^2^ )	25.91 ± 3.41	24.14 ± 3.22	25.79 ± 3.30	26.95 ± 3.30	27.25 ± 3.16	< 0.01
SBP (mmHg)	141.44 ± 19.17	142.03 ± 19.59	141.08 ± 19.21	141.65 ± 18.84	142.73 ± 19.05	< 0.01
DBP (mmHg)	89.31 ± 10.80	88.07 ± 10.81	89.12 ± 10.74	90.08 ± 10.88	91.32 ± 10.63	< 0.01
TC (mmol/L)	5.07 ± 1.35	4.94 ± 0.97	5.02 ± 1.41	5.18 ± 1.04	5.56 ± 2.56	< 0.01
HDL-C (mmol/L)	1.52 ± 0.43	1.91 ± 0.49	1.53 ± 0.38	1.35 ± 0.35	1.19 ± 0.39	< 0.01
LDL-C (mmol/L)	2.62 ± 0.84	2.41 ± 0.83	2.68 ± 0.83	2.66 ± 0.81	2.44 ± 0.98	< 0.01
TG (mmol/L)	1.37 (0.99–2.03)	0.74 (0.59–0.92)	1.26 (1.01–1.60)	2.20 (1.65–2.99)	4.88 (3.53–6.93)	< 0.01
AIP_2006_	-0.04 (-0.44–0.44)	-0.78 (-1.04–-0.54)	-0.16 (-0.41–0.13)	0.57 (0.28–0.90)	1.21 (0.86–1.56)	< 0.01
AIP_2008_	-0.01 (-0.44–0.41)	-0.85 (-1.12–-0.61)	-0.08 (-0.38–0.11)	0.54 (0.23–0.84)	1.36 (1.04–1.76)	< 0.01
AIP_2010_(mg/dl)	-0.07 (-0.47–0.40)	-0.91 (-1.17–-0.67)	-0.17 (-0.42–0.11)	0.53 (0.23–0.84)	1.45 (1.12–1.83)	< 0.01
FPG (mmol/L)	5.86 ± 1.95	5.56 ± 1.32	5.78 ± 1.90	6.10 ± 2.20	6.60 ± 2.32	< 0.01
hs-CRP (mg/L)	1.20 (0.50–2.90)	1.20 (0.63–2.80)	1.10 (0.40–2.70)	1.40 (0.50–3.20)	1.61 (0.78–3.67)	< 0.01
eGFR(ml/1.73m^2^)	84.33 ± 19.43	84.27 ± 18.01	83.79 ± 18.85	84.99 ± 20.80	87.38 ± 22.31	< 0.01
Current smoker, N (%)	7414 (37.0)	971 (33.3)	3931 (35.3)	2079 (41.0)	433 (47.8)	< 0.01
Alcohol drinker, N (%)	6762 (33.8)	925 (31.7)	3499 (31.5)	1917 (37.8)	421 (46.5)	< 0.01
Physical activity, N (%)	3146 (15.7)	495 (17.0)	1785 (16.1)	748 (14.8)	118 (13.0)	< 0.01
Education, N (%)	3912 (19.5)	390 (13.4)	2148 (19.3)	1164 (23.0)	210 (23.2)	< 0.01
Diabetes mellitus, N (%)	3134 (15.7)	264 (9.04)	1542 (13.9)	1058 (20.9)	270 (29.8)	< 0.01
Antihypertensive drugs, N (%)	6161 (30.8)	764 (26.2)	3310 (29.8)	1732 (34.2)	355 (39.2)	< 0.01
Hypoglycemic drugs, N (%)	1601 (8.00)	148 (5.07)	799 (7.18)	521 (10.3)	133 (14.7)	< 0.01
Lipid-lowering drugs, N (%)	271 (1.35)	13 (0.45)	101 (0.91)	130 (2.57)	27 (2.98)	< 0.01

Note: P value, comparison of baseline characteristics between diferent AIP trajectories

BMI body mass index, SBP systolic blood pressure, DBP diastolic blood pressure, HDL-C high-density lipoprotein cholesterol, LDL-C low-density lipoprotein cholesterol, FPG fasting plasma glucose, TG triglyceride, hs-CRP high-sensitivity C reactive protein, AIP atherogenic index of plasma, Education senior high school or above

### Association between AIP trajectories and CVD risk

**Fig. 3:**
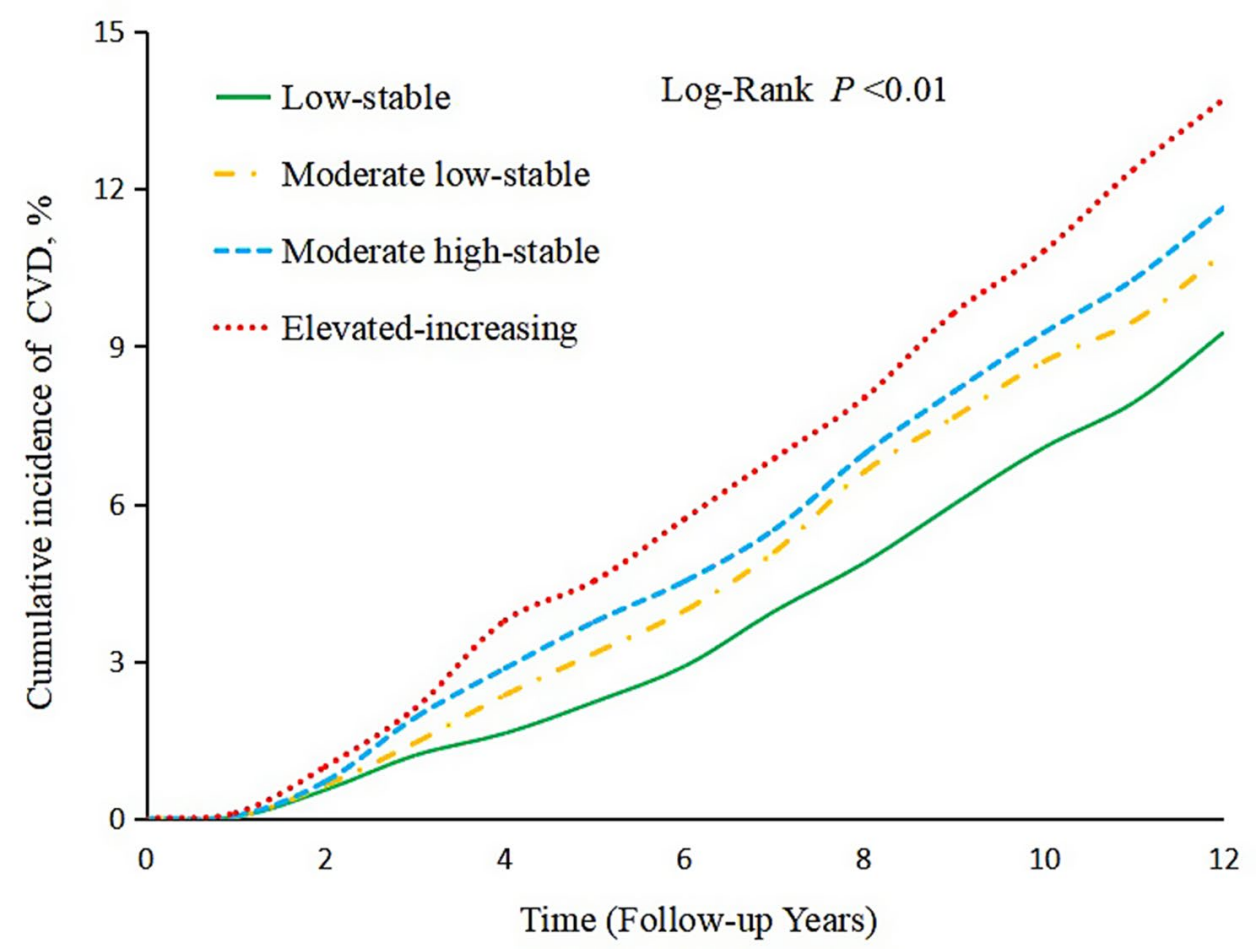
Kaplan-Meier incidence rate of CVD according to AIP trajectories

During a median follow-up period of 11.9 years (interquartile range, 11.3 to 12.3 years), 2,332 (11.65%) participants with hypertension experienced CVD events. There were 444 cases of myocardial infarction, 1,773 cases of ischemic stroke, and 227 cases of hemorrhagic stroke among the participants with hypertension. The cumulative incidence of CVD in the various groups of participants with hypertension differed significantly according to the log-rank test (*P* < 0.01; Fig. 3). Multivariable-adjusted Cox models revealed an increase in CVD risk across ascending AIP trajectory groups (Table 2). Compared with the low-stable group, the moderate high-stable group had a 23% higher CVD risk (HR = 1.23, 95% CI: 1.05–1.44), while the elevated-increasing group exhibited a 43% elevated risk (HR = 1.43, 1.14–1.79; *P*-trend = 0.001). In the subgroup analysis, myocardial infarction risk increased progressively, with the elevated-increasing group showing an 80% higher risk (HR = 1.80, 1.09–2.51; *P*-trend < 0.001). The elevated-increasing group had a 40% increased risk in ischemic stroke (HR = 1.40, 1.08–1.80; *P*-trend = 0.001). No significant associations were observed across AIP trajectories in hemorrhagic stroke (*P*-trend = 0.734) (Table 2).

**Table 2:** The hazard ratios of CVD and subtypes according to trajectories of AIP from 2006 to 2010

AIP trajectories	Case/ Total	IR	Model 1	Model 2	Model 3
	**CVD**				
Low-stable	290/2920	9.23	1.00	1.00	1.00
Moderate low-stable	1263/11,121	10.59	1.26(1.11,1.43)	1.12(0.98,1.29)	1.13(0.99,1.30)
Moderate high-stable	645/5067	11.81	1.49(1.29,1.71)	1.22(1.05,1.43)	1.23(1.05,1.44)
Elevated-increasing	134/905	13.79	1.85(1.51,2.28)	1.46(1.16,1.82)	1.43(1.14,1.79)
*P*-trend			< 0.001	< 0.001	0.001
	**Myocardial infarction**				
Low-stable	46/2920	1.43	1.00	1.00	1.00
Moderate low-stable	230/11,121	1.85	1.45(1.06,1.99)	1.21(0.86,1.70)	1.21(0.86,1.69)
Moderate high-stable	141/5067	2.48	2.09(1.50,2.93)	1.59(1.07,2.38)	1.59(1.07,2.37)
Elevated-increasing	27/905	2.64	2.41(1.49,3.90)	1.83(1.09,2.54)	1.80(1.09,2.51)
*P*-trend			< 0.001	< 0.001	< 0.001
	**Ischemic Stroke**				
Low-stable	226/2920	7.15	1.00	1.00	1.00
Moderate low-stable	962/11,121	7.92	1.21(1.05,1.40)	1.11(0.95,1.29)	1.10(0.94,1.29)
Moderate high-stable	483/5067	8.67	1.41(1.20,1.65)	1.18(1.08,1.28)	1.17(1.08,1.28)
Elevated-increasing	102/905	10.25	1.77(1.49,2.24)	1.41(1.11,1.81)	1.40(1.08,1.80)
*P*-trend			< 0.001	< 0.001	0.001
	**Hemorrhagic stroke**				
Low-stable	34/2920	0.97	1.00	1.00	1.00
Moderate low-stable	129/11,121	0.97	1.05(0.71,1.54)	1.06(0.71,1.60)	1.06(0.70,1.60)
Moderate high-stable	51/5067	0.84	0.94(0.61,1.46)	0.88(0.54,1.45)	0.89(0.54,1.46)
Elevated-increasing	13/905	1.20	1.39(0.73,2.66)	1.12(0.55,2.26)	1.11(0.55,2.27)
*P*-trend			0.784	0.728	0.734

Note: AIP, atherogenic index of plasma; CVD, cardiovascular disease; IR, incidence rate (per 1000 person years)

Model 1: adjusted for age and sex

Model 2: included variables in model 1 and further SBP, LDL-C, BMI, hs-CRP, smoking status, alcohol consumption, physical exercise habits and diabetes mellitus

Model 3: included variables in model 2 and further the use of antihypertensive drugs, the use of hypoglycemic drugs, and the use of lipid-lowering drugs

### Results of the stratified and sensitivity analyses

In stratified analyses, no significant interaction effects were detected across pre-specified subgroups stratified by age, sex, BMI, diabetes status, antihypertensive medication use, or blood pressure control status (all *P* for interaction > 0.05). Elevated AIP trajectories were consistently associated with increased CVD risk in participants aged < 45 and ≥ 45 years, as well as in males, non-obese individuals, those with diabetes, non-diabetes, non-users of antihypertensive drugs, and those with uncontrolled blood pressure. However, no statistically significant associations were observed in females, participants with obesity, antihypertensive drug use, or those achieving blood pressure control (Table 3). Notably, our analyses revealed no significant link between AIP trajectories and hemorrhagic stroke risk, a finding that was robust across all examined subgroups (Table S3).

**Table 3:** Subgroup analyses: the hazard ratios of CVD according to trajectories of AIP from 2006 to 2010

		AIP trajectories				*P*-trend	*P*-interaction
		Low-stable	Moderate low-stable	Moderate high-stable	Elevated-increasing		
Age	Event/Total						0.175
< 45 years	124/2485	1.00	2.32(0.82,6.55)	3.30(1.14,9.55)	3.40(1.03,11.16)	0.014	
≥ 45 years	2208/17,528	1.00	1.14(0.99,1.30)	1.22(1.04,1.43)	1.42(1.13,1.79)	0.001	
Sex							0.660
Female	301/3616	1.00	0.89(0.61,1.23)	0.91(0.56,1.49)	0.94(0.41,2.14)	0.846	
Male	2031/16,397	1.00	1.17(1.01,1.35)	1.26(1.06,1.49)	1.45(1.14,1.85)	0.001	
BMI							0.735
< 28Kg/m^2^	1728/14,256	1.00	1.15(0.96,1.38)	1.27(1.04,1.55)	1.57(1.21,2.03)	< 0.001	
≥ 28Kg/m^2^	604/5757	1.00	1.10(0.89,1.36)	1.27(0.95,1.68)	1.36(0.80,2.32)	0.081	
Diabetes							0.150
Yes	523/3134	1.00	1.40(0.96,2.04)	1.53(1.05,2.11)	2.13(1.35,3.37)	0.005	
No	1809/16,879	1.00	1.09(0.94,1.27)	1.18(0.88,1.56)	1.20(1.02,1.43)	0.034	
Antihypertensive drugs							0.285
Yes	784/6161	1.00	1.10(0.86,1.40)	1.19(0.91,1.58)	1.42(0.98,2.08)	0.055	
No	1548/13,852	1.00	1.15(0.97,1.35)	1.28(1.06,1.55)	1.56(1.18,2.07)	< 0.001	
BP controlled							0.742
Yes	302/4026	1.00	0.92(0.64,1.31)	0.98(0.50,1.93)	1.09(0.71,1.65)	0.558	
No	2030/15,987	1.00	1.17(1.01,1.35)	1.27(1.07,1.51)	1.57(1.24,2.00)	< 0.001	

Note: AIP, atherogenic index of plasma; CVD, cardiovascular disease; BP, blood pressure

Model adjusted for age, sex, SBP, LDL-C, BMI, hs-CRP, smoking status, alcohol consumption, physical exercise habits, diabetes, the use of antihypertensive drugs, the use of hypoglycemic drugs, and the use of lipid-lowering drugs

Sensitivity analyses confirmed the robustness of the primary association. Temporal validation: exclusion of incident CVD cases within 2-year follow-up (*n* = 546); pharmacological confounder control: sequential exclusion of baseline users of antihypertensive (*n* = 6,161), lipid-lowering (*n* = 271), hypoglycemic agents (*n* = 1,592), who newly initiated statin therapy during the follow-up period (*n* = 271). Missing Data Validation: exclusion of participants with missing TC and LDL-C data at baseline (*n* = 405), with AIP values in the highest and lowest 0.5% (extreme outliers) (*n* = 209). Etiological Verification: exclusion of participants with secondary hypertension (*n* = 1,944). Longitudinal covariate adjustment: models incorporating both baseline (2006) and updated (2010) AIP measurements, and included SBP, FBG, BMI, and LDL as time-dependent covariates during the follow-up period. All analyses demonstrated consistent risk estimates, with the detailed results provided in Table S1-S2.

Exploratory analyses demonstrated that both triglyceride-glucose index (TyG) (IDI 0.078%, *P* = 0.008) and AIP (IDI 0.066%, *P* = 0.012) significantly improved CVD risk prediction beyond traditional factors, whereas lipid accumulation product (LAP) and visceral adiposity index (VAI) did not (both *P* > 0.05) (Table S4).

## Discussion

In this prospective cohort study of 20,013 patients with hypertension, we identified four distinct AIP trajectories and demonstrated a dose-dependent relationship between long-term elevated AIP levels and increased CVD risk. Notably, hypertensive individuals in the elevated-increasing AIP trajectory group exhibited a 43% higher risk of CVD than those in the low-stable group, independent of baseline lipid profiles. These associations remained robust across subgroup and sensitivity analyses, highlighting the prognostic value of dynamic AIP monitoring for hypertension management.

While previous studies have established AIP as a predictor of atherosclerosis and CVD in the general population [12, 13], our study extends this evidence to hypertensive patients, a high-risk group with concurrent metabolic dysregulation [20]. Sadeghi et al. demonstrated the independent predictive value of baseline AIP for CVD in the general population [22], which was limited to linear associations between static AIP levels and CVD risk, thus failing to capture the dynamic impact of metabolic fluctuations in high-risk subgroups. In contrast, our study employed Latent Mixture Models to identify four distinct AIP trajectories in a hypertensive cohort, revealing that young hypertensive patients (< 45 years) with rising AIP trajectories exhibited exponential CVD risk escalation (HR = 3.40). This finding underscores the synergistic interplay between blood pressure dysregulation and lipid metabolism disorders in accelerating atherosclerosis. Another AIP trajectory-focused study reported elevated CVD risk in the “increasing trajectory” group, but simplified metabolic dynamics using a two-phase model (declining/increasing) [23]. Our research advances this by analyzing 20,013 hypertensive patients with a median follow-up of 11.9 years, which not only confirmed risk gradients across four trajectories, but also demonstrated that blood pressure control and antihypertensive medication significantly attenuated AIP trajectory-associated risks. This is the first study to elucidate the synergistic protective effects of dynamic blood pressure-lipid management in a hypertensive cohort, bridging a critical gap in high-risk population stratification. Importantly, unlike prior studies that relied on single AIP measurements [24, 25], our trajectory analysis revealed that persistent AIP elevation rather than transient fluctuation drives CVD risk, aligning with the chronicity of atherosclerotic progression. Furthermore, in exploratory comparative analyses using the average of three measurements (2006–2010), both the TyG and AIP demonstrated significant improvements in model performance [26, 27]. In contrast, LAP and VAI did not achieve statistically significant improvements. These findings further underscore that while static single-timepoint indices offer modest predictive value, the dynamic AIP trajectory approach captures cumulative atherogenic risk over time, a dimension that cross-sectional comparisons cannot address.

Subgroup analyses revealed a pronounced CVD risk associated with elevated AIP trajectories in younger hypertensive patients (< 45 years: HR = 3.40 vs. ≥45 years: HR = 1.42), although the test for interaction was not statistically significant. This finding mirrors the rising burden of early onset hypertension and metabolic syndrome globally [28–30]. Mechanistically, younger hypertensive individuals often exhibit a dyslipidemia triad (high TG, low HDL-C, and elevated LDL-C), which accelerates endothelial dysfunction and plaque instability [28, 31, 32]. AIP magnifies these imbalances, making it a sensitive marker for subclinical atherosclerosis in younger populations [33]. Clinically, our results advocate for routine AIP screening in young hypertensive patients to identify high-risk subgroups warranting aggressive lipid management [34, 35].

AIP offers a cost-effective and noninvasive tool for CVD risk stratification in hypertensive populations. Our findings support the integration of the AIP into routine hypertension care, particularly in younger patients with metabolic comorbidities. Lifestyle interventions and Statin therapy can effectively lower AIP [36, 37], potentially mitigating CVD risk. Public health initiatives should emphasize early AIP monitoring alongside blood pressure control to curb the dual epidemics of hypertension and dyslipidemia.

The relationship between elevated AIP levels and cardiovascular disease risk may involve interrelated pathophysiological mechanisms. Elevated AIP reflects an atherogenic lipid profile characterized by increased levels of small, dense LDL particles, which exhibit heightened oxidative susceptibility and capacity to infiltrate arterial walls, thereby promoting inflammatory responses and plaque destabilization [38–40]. Concurrently, a high AIP correlates with impaired HDL functionality, particularly reduced cholesterol esterification rates that compromise reverse cholesterol transport efficiency, leading to vascular lipid accumulation and accelerated atherogenesis [38, 41, 42]. These lipid-driven effects synergize with broader metabolic dysregulation, as AIP-associated lipid abnormalities exacerbate insulin resistance and endothelial dysfunction, creating a self-reinforcing cycle that potentiates hypertension and vascular remodeling, ultimately amplifying cardiovascular risk [11, 38, 43]. Therefore, the four distinct AIP trajectories we identified likely represent subgroups of hypertensive patients with varying intensities of these underlying pathophysiological processes, providing a biological continuum that explains the gradient in CVD risk [44, 45].

The strengths of this study include its large-scale longitudinal design, rigorous biennial follow-ups, and novel application of trajectory modeling to AIP dynamics. However, this study has some limitations. First, residual confounding may persist despite multivariate adjustments. Second, our cohort was predominantly northern Chinese, with a higher proportion of males, limiting generalizability to other demographics. Third, we could not effectively distinguish participants with primary hypertension from those with secondary hypertension [46]. Fourth, although we employed a latent mixture model to identify AIP trajectories, the classification remains data-driven and may not fully capture the biological complexity of individual lipid metabolism variations. While the model demonstrated good statistical fit and biological plausibility, some subtle or non-linear trajectory patterns might have been overlooked. This inherent limitation of trajectory modeling approaches should be considered when interpreting the results. Finally, the observational design could not establish causal inferences; thus, future studies should address [47].

## Conclusions

In this large-scale longitudinal study of patients with hypertension, long-term elevation in AIP trajectories was independently associated with an increased risk of CVD, particularly myocardial infarction and ischemic stroke. Persistent AIP trajectory monitoring enables the identification of hypertensive individuals with a heightened cardiovascular risk. Sustained maintenance of optimal AIP levels, combined with blood pressure control and antihypertensive medication adherence, reduced CVD incidence.

## Supplementary Information

Below is the link to the electronic supplementary material.

Supplementary Material 1

## Data Availability

The datasets used and analyzed during the current study are available from the corresponding author upon reasonable request.

## Abbreviations

AIP: Atherogenic Index of Plasma
CVD: Cardiovascular disease
TG: Triglyceride
FPG: Fasting plasma glucose
BMI: Body mass index
BP: Blood pressure
SBP: Systolic blood pressure
DBP: Diastolic blood pressure
TC: Total cholesterol
HDL-C: High-density lipoprotein cholesterol
hs-CRP: High-sensitivity C-reactive protein
LDL-C: Low-density lipoprotein cholesterol
LAP: Lipid accumulation product
VAI: Visceral adiposity index
HR: Hazard ratio
CI: Confidence interval
