# Supplementary material for: Association between the atherogenic index of plasma trajectory and risk of cardiovascular diseases among hypertensive patients: a prospective cohort study
Source: Cardiovasc Diabetol Endocrinol Rep. 2026 Jun 12;12:32. doi: 10.1186/s40842-026-00302-7 (PMC13262429; doi:10.1186/s40842-026-00302-7)
Supplement: Supplementary file 1 — Supplementary Material 1 [file 40842_2026_302_MOESM1_ESM.docx]

**Additional file**

**Table S1** Sensitivity analysis for association of AIP trajectories and CVD risks

| AIP trajectories | Analysis 1 | Analysis 2 | Analysis 3 | Analysis 4 | Analysis 5 | Analysis 6 |
| --- | --- | --- | --- | --- | --- | --- |
| Low-stable | 1.00 | 1.00 | 1.00 | 1.00 | 1.00 | 1.00 |
| Moderate low-stable | 1.13(0.97,1.30) | 1.15(0.97,1.35) | 1.14(1.00,1.30) | 1.15(0.99,1.31) | 1.12(0.97,1.29) | 1.10(0.96,1.26) |
| Moderate high-stable | 1.23(1.04,1.46) | 1.28(1.06,1.55) | 1.23(1.05,1.45) | 1.24(1.05,1.44) | 1.22(1.03,1.41) | 1.21(1.02,1.40) |
| Elevated-increasing | 1.40(1.10,1.79) | 1.56(1.18,2.07) | 1.45(1.16,1.82) | 1.44(1.15,1.80) | 1.41(1.14,1.69) | 1.40(1.12,1.68) |
| *P*-trend | 0.002 | <0.001 | <0.001 | 0.001 | 0.001 | 0.002 |

Note: AIP, atherogenic index of plasma; CVD, cardiovascular disease.

Analysis 1：Excluded participants who developed CVD within 2 year of follow-up (n=546).

Analysis 2：Excluded participants with antihypertensive drugs at baseline (n=6161).

Analysis 3：Excluded participants with lipid-lowering drugs at baseline (n=271).

Analysis 4：Excluded participants with hypoglycemic drugs at baseline (n=1592).

Analysis 5：Sensitivity analysis of additionally adjusted for AIP in 2006

Analysis 6：Sensitivity analysis of additionally adjusted for AIP in 2010

Analysis 1: Model adjusted for age, sex, SBP, LDL-C, BMI, hs-CRP, smoking status, alcohol consumption, physical exercise habits, diabetes, the use of antihypertensive drugs, the use of hypoglycemic drugs, and the use of lipid-lowering drugs.

Analysis 2：Model adjusted for age, sex, SBP, LDL-C, BMI, hs-CRP, smoking status, alcohol consumption, physical exercise habits, diabetes, the use of hypoglycemic drugs, and the use of lipid-lowering drugs.

Analysis 3：Model adjusted for age, sex, SBP, LDL-C, BMI, hs-CRP, smoking status, alcohol consumption, physical exercise habits, diabetes, the use of antihypertensive drugs, and the use of hypoglycemic drugs.

Analysis 4：Model adjusted for age, sex, SBP, LDL-C, BMI, hs-CRP, smoking status, alcohol consumption, physical exercise habits, diabetes, the use of antihypertensive drugs, and the use of lipid-lowering drugs.

Analysis 5: Model adjusted for age, sex, SBP, LDL-C, BMI, hs-CRP, smoking status, alcohol consumption, physical exercise habits, diabetes, the use of antihypertensive drugs, the use of hypoglycemic drugs, the use of lipid-lowering drugs, and AIP in 2006.

Analysis 6: Model adjusted for age, sex, SBP, LDL-C, BMI, hs-CRP, smoking status, alcohol consumption, physical exercise habits, diabetes, the use of antihypertensive drugs, the use of hypoglycemic drugs, the use of lipid-lowering drugs, and AIP in 2010.

**Table S2** Sensitivity analysis of the association between AIP trajectories and CVD risks

| **AIP trajectories** | **Analysis 1** | **Analysis 2** | **Analysis 3** | **Analysis 4** | **Analysis 5** |
| --- | --- | --- | --- | --- | --- |
| Case/Total | 2094/18069 | 2303/19742 | 2284/19608 | 2317/19804 | 2332/20013 |
| Low-stable | 1.00 | 1.00 | 1.00 | 1.00 | 1.00 |
| Moderate low-stable | 1.08(0.94,1.24) | 1.14(0.99,1.31) | 1.14(0.99,1.31) | 1.14(0.99,1.31) | 1.12(1.05,1.20) |
| Moderate high-stable | 1.16(0.99,1.37) | 1.24(1.06,1.45) | 1.23(1.05,1.45) | 1.23(1.05,1.44) | 1.28(1.19,1.38) |
| Elevated-increasing | 1.37(1.09,1.74) | 1.46(1.17,1.83) | 1.45(1.16,1.82) | 1.45(1.15,1.82) | 1.50(1.35,1.68) |
| *P*-trend | 0.006 | <0.001 | <0.001 | 0.001 | 0.001 |

Note: AIP, atherogenic index of plasma; CVD, cardiovascular disease.

Analysis 1：Excluded participants with secondary hypertension (n=1944).

Analysis 2：Excluded participants who newly initiated statin therapy during the follow-up period (n=271).

Analysis 3：Excluded participants with missing TC and LDL-C data at baseline (n=405).

Analysis 4：Excluded participants with AIP values in the highest and lowest 0.5% (extreme outliers) (n=209).

Analysis 5：Included SBP, FBG, BMI, and LDL as time-dependent covariates during the follow-up period.

Analysis 1: Model adjusted for age, sex, SBP, LDL-C, BMI, hs-CRP, smoking status, alcohol consumption, physical exercise habits, diabetes, the use of antihypertensive drugs, the use of hypoglycemic drugs, and the use of lipid-lowering drugs.

Analysis 2：Model adjusted for age, sex, SBP, LDL-C, BMI, hs-CRP, smoking status, alcohol consumption, physical exercise habits, diabetes, the use of antihypertensive drugs, the use of hypoglycemic drugs, and the use of lipid-lowering drugs.

Analysis 3：Model adjusted for age, sex, SBP, BMI, hs-CRP, smoking status, alcohol consumption, physical exercise habits, diabetes, the use of antihypertensive drugs, the use of hypoglycemic drugs, and the use of lipid-lowering drugs.

Analysis 4：Model adjusted for age, sex, SBP, LDL-C, BMI, hs-CRP, smoking status, alcohol consumption, physical exercise habits, diabetes, the use of antihypertensive drugs, the use of hypoglycemic drugs, and the use of lipid-lowering drugs.

Analysis 5: Model adjusted for SBP, FBG, BMI, and LDL as time-dependent covariates, age, sex, hs-CRP, smoking status, alcohol consumption, physical exercise habits, the use of antihypertensive drugs, the use of hypoglycemic drugs, the use of lipid-lowering drugs at baseline.

**Table S3 Subgroup analyses: the hazard ratios of hemorrhagic stroke according to trajectories of AIP from 2006 to 2010**

|  |  | AIP trajectories | | | | *P*-trend | *P*-interaction |
| --- | --- | --- | --- | --- | --- | --- | --- |
|  |  | Low-stable | Moderate low-stable | Moderate high-stable | Elevated-increasing |  |  |
| Age | Event/Total |  |  |  |  |  | 0.944 |
| <45 years | 16/2485 | 1.00 | 0.40(0.07,2.32) | 0.58(0.09,3.81) | 0.43(0.03,6.27) | 0.922 |  |
| ≥ 45 years | 211/17528 | 1.00 | 1.11(0.73,1.69) | 0.87(0.52,1.46) | 1.16(0.56,2.41) | 0.692 |  |
| Sex |  |  |  |  |  |  | 0.788 |
| Female | 31/3616 | 1.00 | 1.87(0.58,6.03) | 1.64(0.38,7.07) | 1.54(0.14,16.57) | 0.690 |  |
| Male | 196/16397 | 1.00 | 0.99(0.64,1.53) | 0.83(0.49,1.40) | 1.07(0.51,2.25) | 0.664 |  |
| BMI |  |  |  |  |  |  | 0.364 |
| <28Kg/m^2^ | 164/14256 | 1.00 | 0.62(0.34,1.15) | 0.73(0.31,1.70) | 0.41(0.05,3.24) | 0.283 |  |
| ≥28Kg/m^2^ | 63/5757 | 1.00 | 1.71(0.92,3.19) | 1.32(0.66,2.62) | 1.84(0.78,4.36) | 0.768 |  |
| Diabetes |  |  |  |  |  |  | 0.324 |
| Yes | 48/3134 | 1.00 | 2.96(0.67,12.93) | 2.91(0.62,13.54) | 3.21(0.57,18.02) | 0.368 |  |
| No | 179/16879 | 1.00 | 0.91(0.59,1.41) | 0.71(0.41,1.22) | 0.92(0.39,2.14) | 0.312 |  |
| Antihypertensive drugs |  |  |  |  |  |  | 0.486 |
| Yes | 75/6161 | 1.00 | 1.12(0.53,2.38) | 1.03(0.43,2.47) | 0.68(0.16,2.75) | 0.634 |  |
| No | 152/13852 | 1.00 | 1.06(0.65,1.73) | 0.83(0.45,1.53) | 1.44(1.63,3.27) | 0.990 |  |
| BP controlled |  |  |  |  |  |  | 0.641 |
| Yes | 17/4026 | 1.00 | 1.57(0.31,7.96) | 1.10(0.15,8.22) | 0.00(0.00) | 0.686 |  |
| No | 210/15987 | 1.00 | 1.01(0.66,1.54) | 0.85(0.51,1.41) | 1.11(0.54,2.28) | 0.719 |  |

Note: AIP, atherogenic index of plasma; CVD, cardiovascular disease; BP, blood pressure.

Model adjusted for age, sex, SBP, LDL-C, BMI, hs-CRP, smoking status, alcohol consumption, physical exercise habits, diabetes, the use of antihypertensive drugs, the use of hypoglycemic drugs, and the use of lipid-lowering drugs.

**Table S4** Comparison of predictive ability for CVD between AIP, LAP, TyG and VAI

|  | **C-index** | **NRI, (%)** |  | **IDI, (%)** |  |
| --- | --- | --- | --- | --- | --- |
|  | **Estimate (95% CI)** | **Estimate (95% CI), %** | **P-value** | **Estimate (95% CI), %** | **P-value** |
| Base model* | 0.6313 | Ref. |  | Ref. |  |
| Base model + AIP | 0.6334 | 7.13 (2.82, 11.44) | <0.001 | 0.066 (0.03, 0.11) | 0.022 |
| Base model + LAP | 0.6326 | 6.33 (2.12, 10.54) | <0.001 | 0.041 (0.01, 0.08) | 0.066 |
| Base model + TyG | 0.6336 | 9.22 (4.91, 13.53) | <0.001 | 0.078 (0.03, 0.12) | 0.011 |
| Base model + VAI | 0.6321 | 5.21 (0.89, 9.52) | <0.001 | 0.016 (0.00, 0.03) | 0.078 |

NRI, net reclassification index. IDI, integrated discrimination improvement. LAP, Lipid Accumulation Product. TyG, Triglyceride-Glucose Index. VAI, Visceral Adiposity Index.

*Base model was adjusted for age, sex, SBP, LDL-C, BMI, hs-CRP, smoking, alcohol consumption, physical activity, and use of antihypertensive, lipid-lowering, and glucose-lowering medications.
